# *Streptococcus pneumoniae *induced c-Jun-N-terminal kinase- and AP-1 -dependent IL-8 release by lung epithelial BEAS-2B cells

**DOI:** 10.1186/1465-9921-7-98

**Published:** 2006-07-12

**Authors:** Bernd Schmeck, Kerstin Moog, Janine Zahlten, Vincent van Laak, Philippe Dje N'Guessan, Bastian Opitz, Simone Rosseau, Norbert Suttorp, Stefan Hippenstiel

**Affiliations:** 1 Department of Internal Medicine/Infectious Diseases and Respiratory Medicine, Charité – Universitätsmedizin Berlin, 13353 Berlin, Germany; 2 Department of Peridontology and Synoptic Dentistry, Charité – Universitätsmedizin Berlin, 13353 Berlin, Germany

## Abstract

**Background:**

Although pneumococcal pneumonia is one of the most common causes of death due to infectious diseases, little is known about pneumococci-lung cell interaction. Herein we tested the hypothesis that pneumococci activated pulmonary epithelial cell cytokine release by c-Jun-NH_2_-terminal kinase (JNK)

**Methods:**

Human bronchial epithelial cells (BEAS-2B) or epithelial HEK293 cells were infected with *S. pneumoniae *R6x and cytokine induction was measured by RT-PCR, ELISA and Bioplex assay. JNK-phosphorylation was detected by Western blot and nuclear signaling was assessed by electrophoretic mobility shift assay (EMSA) and chromatin immunoprecipitation (ChIP). JNK was modulated by the small molecule inhibitor SP600125 and AP1 by transfection of a dominant negative mutant.

**Results:**

*S. pneumoniae *induced the release of distinct CC and CXC, as well as Th1 and Th2 cytokines and growth factors by human lung epithelial cell line BEAS-2B. Furthermore, pneumococci infection resulted in JNK phosphorylation in BEAS-2B cells. Inhibition of JNK by small molecule inhibitor SP600125 reduced pneumococci-induced IL-8 mRNA expression and release of IL-8 and IL-6. One regulator of the *il8 *promoter is JNK-phosphorylated activator protein 1 (AP-1). We showed that *S. pneumoniae *time-dependently induced DNA binding of AP-1 and its phosphorylated subunit c-Jun with a maximum at 3 to 5 h after infection. Recruitment of Ser^63/73^-phosphorylated c-Jun and RNA polymerase II to the endogenous *il8 *promoter was found 2 h after *S. pneumoniae *infection by chromatin immunoprecipitation. AP-1 repressor A-Fos reduced IL-8 release by TLR2-overexpressing HEK293 cells induced by pneumococci but not by TNFα. Antisense-constructs targeting the AP-1 subunits Fra1 and Fra2 had no inhibitory effect on pneumococci-induced IL-8 release.

**Conclusion:**

*S. pneumoniae*-induced IL-8 expression by human epithelial BEAS-2B cells depended on activation of JNK and recruitment of phosphorylated c-Jun to the *il8 *promoter.

## Background

Pneumonia is the most common cause of death due to infectious diseases in industrialized countries [1]. Over 40 % of all cases are due to *Streptococcus pneumoniae*, which is the most frequent etiologic agent of community-acquired pneumonia [2,3]. Despite the availability of vaccines and antibiotic treatments, mortality rates remain high [2,4]. Importantly, the number of antibiotic resistant strains is increasing and even vancomycin-tolerant strains have been observed [5].

Cytokine liberation and subsequent recruitment and activation of leucocytes are a hallmark in pneumococci pneumonia usually leading to elimination of the pathogens. Although immune cells like alveolar macrophages significantly contribute to the activation of the host immune system, evidence has been presented that lung epithelium considerably participates in the recognition of invading pathogens and initiation of the host response [6]. Since the pulmonary epithelium constitutes a large surface, which is in direct contact with invading pathogens, analysis of the interaction between pathogens and pulmonary epithelial cells is of considerable interest.

Host cell activation by *S. pneumoniae *involved membrane-bound pattern recognition receptors TLR2 [7,8]and TLR4 [8,9]. Moreover, we recently demonstrated that cytosolic Nod2 protein [10] recognized invading, cytosolic pneumococci. Pneumococci infection of lung epithelial cells initiated complex signaling pathways leading to activation of the canonical NF-κB pathway and subsequent expression of pro-inflammatory genes. Activation of mitogen-activated protein kinase (MAPK) pathways participated in lung cell activation by pneumococci. For example, p38 MAPK activation induced phosphorylation of NF-κB p65/RelA at serine 536 at the interleukin-8 (IL-8) promoter thus paving the way for RNA polymerase II recruitment, and subsequent IL-8 transcription in pneumococci infected epithelium [11]. In addition, stimulation of c-Jun N-terminal kinase/stress-activated protein kinase JNK/SAPK kinase was shown in pneumococci infected cells [12]. In other model systems, JNK was shown to subsequently activate transcription factor activator protein-1 (AP-1) [13], a central regulator of cytokine expression, by phosphorylating its component c-Jun on serine 63 and serine 73 in the NH_2_-terminal activation domain [14,15].

In this study, we analyzed the liberation of different cytokines families as well as of growth factors by pneumococci infected BEAS-2B cells and tested the role of the JNK kinase pathway for cytokine liberation by using IL-8 as a model cytokine.

Pneumococci induced liberation of a broad array of chemo- and cytokines as well as growth factors. *S. pneumoniae *infection resulted in JNK phosphorylation, and increased AP-1-DNA-binding in BEAS-2B cells. Inhibition of JNK reduced pneumococci-induced IL-8 mRNA expression and release of IL-8 and IL-6. In addition, recruitment of Ser^63/73^-phosphorylated c-Jun and RNA polymerase II to the endogenous *il8 *promoter was found after *S. pneumoniae *infection by chromatin immunoprecipitation. AP-1 repressor A-Fos reduced IL-8 release induced by pneumococci but not by TNFα. In contrast, antisense-constructs targeting the AP-1 subunits Fra1 and Fra2 had no inhibitory effect on pneumococci-induced IL-8 release. In conclusion, JNK-and AP-1-dependent activation of lung epithelial BEAS-2B cells lead to expression of IL-8.

## Materials and methods

### Materials

DMEM, FCS, trypsin-EDTA-solution, CA-650, and antibiotics were obtained from Life Technologies (Karlsruhe, Germany). TNFα was purchased from R&D Systems (Wiesbaden, Germany). All other chemicals used were of analytical grade and obtained from commercial sources.

### Cell lines

Human bronchial epithelial BEAS-2B cells were a kind gift of C. Harris (NIH, Bethesda, MD) [16]. Human embryonic kidney cells (HEK293) were purchased from ATCC (Rockville, USA).

### Bacterial strains

*S. pneumoniae *R6x is the unencapsulated derivative of type 2 strain D39 [17]. Single colony isolates of R6x were maintained at 37°C with 5% CO_2 _on Columbia agar with 5% sheep blood. For cell culture stimulation studies, single colonies were expanded by resuspension in Todd-Hewitt broth supplemented with 0.5% yeast extract and incubation at 37°C for 3 – 4 h to midlog phase (A_600 _0.2 – 0.4), harvested by centrifugation and resuspended in cell culture medium at the indicated concentration without antibiotics as described [11]. Cell viability was validated by microscopy and measurement LDH release into the supernatant.

### Plasmids, and transient transfection procedures

HEK293 cells were cultured in 12-well plates with DMEM supplemented with 10% FCS. Subconfluent cells were co-transfected by using Superfect (Qiagen, Hilden, Germany) according to the manufacturer's instructions (Clonetech, Palo Alto, USA) with 0.1 μg of hTLR2 (generously provided by Tularik Inc., San Francisco, USA [18]) and dominant-negative A-Fos (kind gift of Dr. Charles Vinson, NCI, NIH, Rockville, MD) [19], or Fra1- or Fra2-antisense (kind gift of Dr. Vladimir Berezin, Institute of Molecular Pathology, School of Medicine, Copenhagen University, Copenhagen, Denmark) [20] expression vectors or control vector. Cells were incubated with R6x for 6 h.

### IL-6 and IL-8 ELISA

Confluent BEAS-2B cells were stimulated for 15 h in a humidified atmosphere. After incubation supernatants were collected. In some experiments, cells were lysed with mellitin for 30 min [21]. Supernatants and lysates were processed for IL-6 or IL-8-quantification by sandwich-ELISA as described previously [8].

### Bioplex protein array system

Confluent BEAS-2B cells were infected for 15 h with pneumococci as indicated in a humidified atmosphere. After incubation supernatants were collected and cytokine release was analyzed with the Bioplex Protein Array system (BioRad, Hercules, CA) using beads specific for IL-2, IL-4, IL-5, IL-6, IL-7, IL-8, IL-10, IL-12 (p70), IL-13, IL-17, MCP-1, TNFα, IL-1β, IFNγ, GM-CSF and MIP-1β, according to the manufacturers instructions as described previously [20].

### RT-PCR

For analysis of IL-8 and GAPDH gene expression in BEAS-2B cells total RNA was isolated with RNEasy Mini kit (Quiagen, Hilden, Germany) and reverse transcribed using AMV reverse transcriptase (Promega, Heidelberg, Germany). Generated cDNA was amplified by PCR using specific intron-spanning specific primers for IL-8 and GAPDH. All primers were purchased from TIB MOLBIOL, Berlin, Germany. After 35 amplification cycles, PCR products were analyzed on 1.5 % agarose gels, stained with ethidium bromide and subsequently visualized. To confirm use of equal amounts of RNA in each experiment, all samples were checked for GAPDH mRNA expression [11].

### Western Blot

For determination of JNK phosphorylation, BEAS-2B cells were infected as indicated, washed twice, and harvested. Cells were lysed in buffer containing Triton X-100, subjected to SDS-PAGE and blotted on Hybond-ECL membrane (Amersham Biosciences, Freiburg, Germany). Immunodetection of phosphorylated JNK was carried out with phospho-specific JNK antibody (Cell Signaling, Frankfurt, Germany) [12]. In all experiments, actin (Santa Cruz Biotechnologies, Santa Cruz, CA) was detected simultaneously to confirm equal protein load. Proteins were visualized by incubation with secondary IRDye 800- or Cy5.5-labeled antibodies, respectively, and quantified by Licor Odyssey software (Odyssey infrared imaging system, LI-COR Inc.) [10,11].

### Electrophoretic mobility shift assay (EMSA)

After stimulation of BEAS-2B cells nuclear protein was isolated and analyzed by EMSA as described previously [22-24]. IRDye800-labeled consensus AP-1 oligonucleotides (GTC AGT CAG TGA CTC AAT CGG TCA) were purchased from Metabion, Planegg-Martinsried, Germany. Briefly, EMSA binding reactions were performed by incubating 7.5 μg of nuclear extract with the annealed oligos according to the manufacturer's instructions. The reaction mixture was subjected to electrophoresis on a 5% native gel and analyzed by Odyssey infrared imaging system (LI-COR Inc.).

### P-c-Jun Transcription factor assay assay (Trans AM™)

The P-c-Jun TransAM™ Assay (Active Motif, Carlsbad, CA) was used to detect DNA binding of P-c-Jun containing AP-1 dimers according to the manufacturer's instructions. Briefly, BEAS-2B cells were stimulated, and 10 μg of nuclear cell extract (containing activated transcription factor) were given in oligonucleotide-coated wells. After 20 min of incubation at room temperature with mild agitation, the plate was washed, and 100 μl/well of the diluted P-c-Jun antibody (1:1000) was incubated for 1 h. The plate was washed 3 times and 100 μl HRP-conjugated antibody (1:1000) was added for 1 h. Developing solution was incubated for 10 min. The reaction was stopped and absorbance was read at 450 nm.

### Chromatin immunoprecipitation

BEAS-2B cells were stimulated, culture medium was removed and 1% formaldehyde was added. After 1 min, cells were washed in ice-cold 0.125 M glycin in PBS and then rapidly collected in ice cold PBS, centrifuged and washed twice with ice cold PBS as described previously [11]. Cells were lysed in RIPA buffer (10 mM Tris (pH 7.5), 150 mM NaCl, 1% NP-40, 1% desoxycholic acid, 0.1% SDS, 1 mM EDTA, 1% aprotinin) and the chromatin was sheared by sonication. Lysates were cleared by centrifugation and supernatants were stored in aliquots at -80°C until further use. Antbodies were purchased from Santa Cruz Biotechnology, Santa Cruz, CA (P-c-Jun and Pol II). Immunoprecipitations from soluble chromatin were carried out overnight at 4°C. Immune complexes were collected with protein A/G agarose for 60 min and washed twice with RIPA Buffer, once with high-salt buffer (2 M NaCl, 10 mM Tris pH 7.5, 1% NP-40, 0.5% desoxycholic acid, 1 mM EDTA) followed by another wash in RIPA Buffer and one wash with TE Buffer (10 mM Tris (pH 7.5), 1 mM EDTA). Immune complexes were extracted in elution buffer (1 TE Buffer containing 1% SDS) by shaking the lysates for 15 min at 1200 rpm, 30°C. They were then digested with RNAse (1 μg/20 μl) for 30 min at 37°C. After proteinase K digestion (1 μg/8 μl for 6 h at 37°C and 6 h at 65°) DNA was extracted using a PCR purification kit (Qiagen, Hilden, Germany). *il8 *promoter DNA was amplified by PCR using Hotstart Taq (Qiagen) polymerase. The PCR conditions were 95°C for 15 min, 33 – 35 cycles of 94°C for 20 s, 60°C for 20 s, 72°C for 20 s. PCR products were separated by agarose gel electrophoresis and detected by ethidium bromide staining. Equal amounts of input DNA was controlled by gel electrophoresis.

The following *il8 *promoter-specific primers were used: sense 5'-AAG AAA ACT TTC GTC ATA CTC CG-3'; antisense 5'-TGG CTT TTT ATA TCA TCA CCC TAC-3' [11].

### Statistical methods

**Figure 1:**
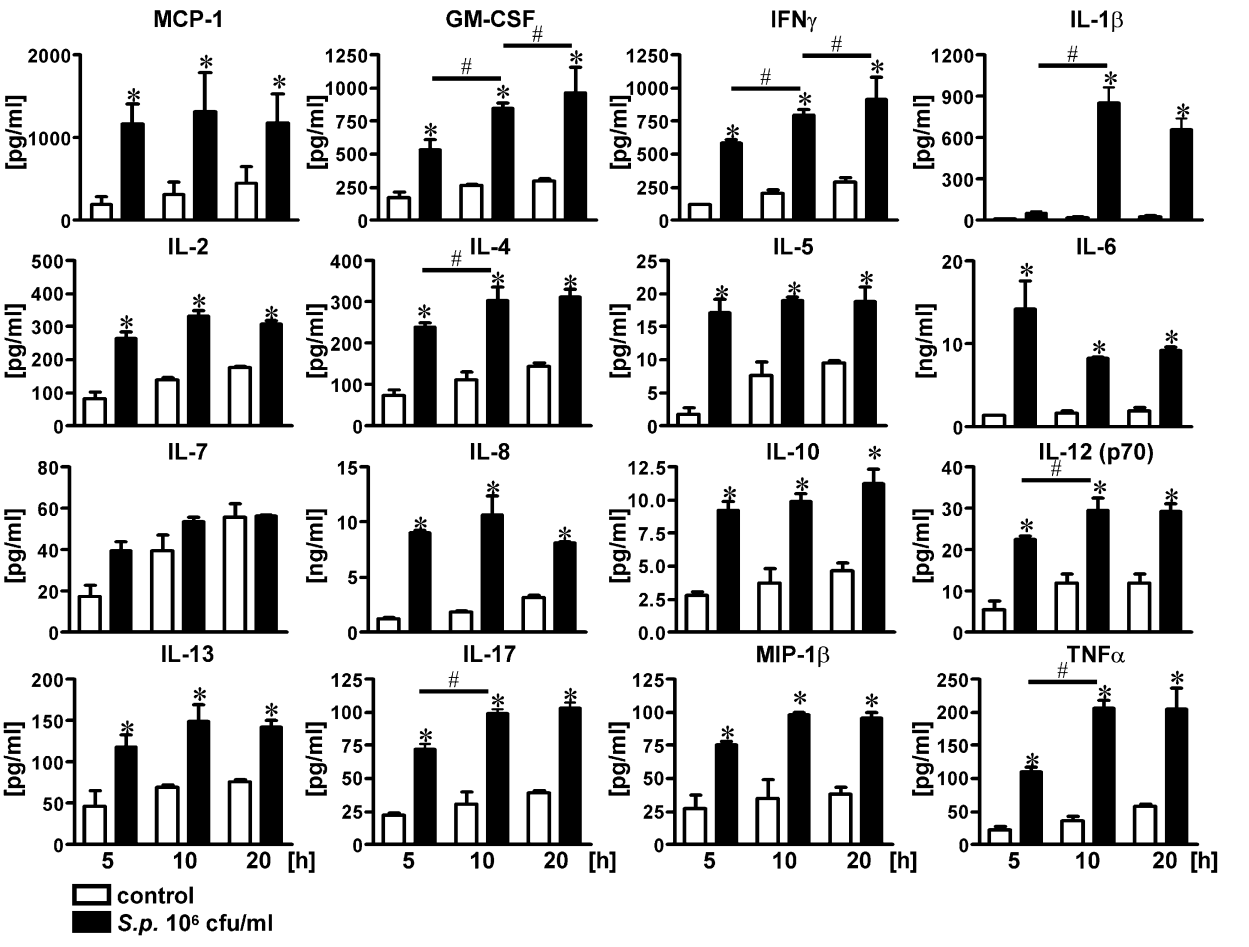
*S. pneumoniae *induce the release of distinct CC and CXC, as well as Th1 and Th2 cytokines and growth factors by human lung epithelial cells. BEAS-2B cells were infected with *S. pneumoniae *strain R6x (10^6 ^cfu/ml) for 5, 10, or 20 h. Cytokine release in the supernatant was measured by Bioplex assay. *, p < 0.01 vs. uninfected control, #, p < 0.01 one time point vs. another, at least in three independent experiments.

**Figure 2:**
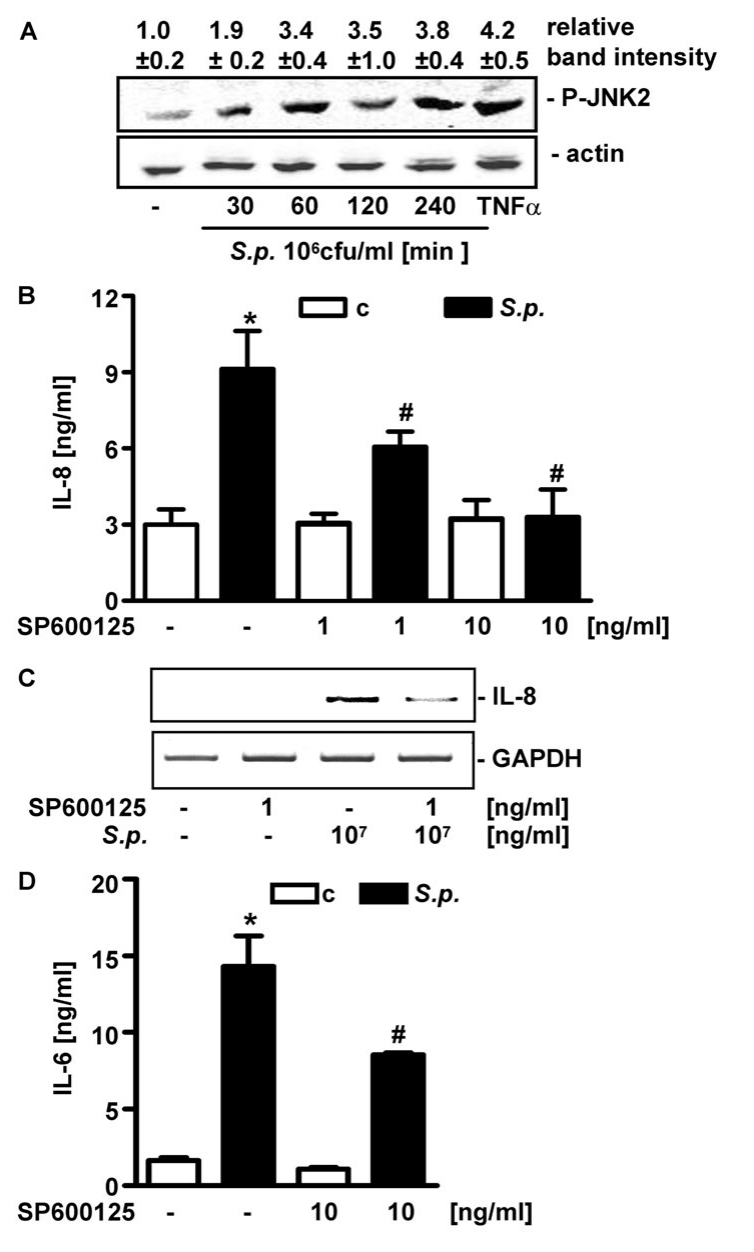
*S. pneumoniae *induced JNK-dependent IL-8 and IL-6 release by human lung epithelial cells. (A) BEAS-2B cells were infected with 10^6 ^cfu/ml *S. pneumoniae *R6x for the times and JNK2 phosphorylation was detected by Western blot. A representative of three independent experiments is shown and quantification of all three experiments is given. (B/D) BEAS-2B cells were preincubated with the indicated concentrations of JNK inhibitor SP600125 and then infected with 10^6 ^cfu/ml *S. pneumoniae *R6x for 15 h. IL-8 (B) and IL-6 (D) concentrations were measured in the supernatant. *, p < 0.01 vs. control; #, p < 0.01 vs. infected cells without pre-incubation with inhibitors in three independent experiments. (C) BEAS-2B cells were preincubated with the indicated concentrations of JNK inhibitor SP600125 and then infected with 10^6 ^cfu/ml *S. pneumoniae *R6x for 3 h. IL-8 and GAPDH mRNA was detected by RT-PCR. Representative gels of three independent experiments are shown.

**Figure 3:**
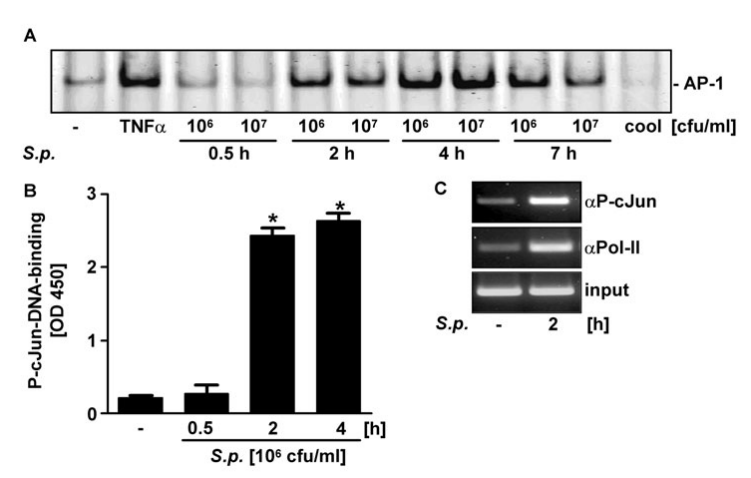
*S. pneumoniae *induced AP-1 activation in human lung epithelial cells. BEAS-2B cells were infected with *S. pneumoniae *R6x (10^6 ^or 10^7 ^cfu/ml as indicated) (A/B/C) for the indicated times or TNFα (50 ng/ml, 0.5 h). DNA binding of AP-1 (A) was detected by EMSA and of phosphorylated c-Jun by transcription factor activation assay (B). *, p < 0.01 vs. control. Recruitment of Ser^63/73^-phosphorylated c-Jun and RNA polymerase II to the endogenous *il8 *promoter was detected by chromatin immunoprecipitation (C). Representatives of at least three independent experiments are shown.

Data are shown as means ± SEM of at least three independent experiments. A one-way ANOVA was used for data of Fig. 1, 2B, 2D, 3B, and 4. Data are shown as means ± SEM of at least three separate experiments. Main effects were then compared by a Newman-Keuls' post-test. P < 0.01 was considered to be significant and indicated by asterisks.

**Figure 4:**
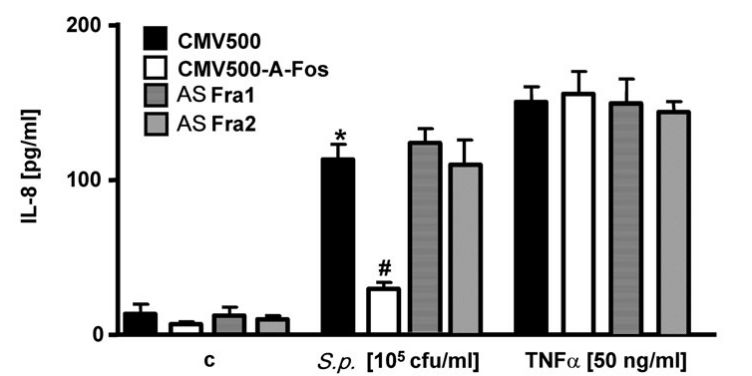
AP-1 repressor blocked *S. pneumoniae*-induced IL-8 release. HEK293 cells were transfected with plasmids encoding TLR2, as well as empty vector (CMV500), AP-1 repressor (CMV500-A-Fos), Fra1 antisense (AS Fra1), or Fra2 antisense (AS Fra2), respectively. Then, cells were infected with *S. pneumoniae *strain R6x (10^5 ^cfu/ml) or stimulated with TNFα (50 ng/ml) for 15 h, and IL-8 concentration was measured in the supernatant. *, p < 0.01 vs. control; #, p < 0.01 vs. infected cells with empty vector at least in three independent experiments.

## Results

### *S. pneumoniae *induced cytokine release in human lung epithelial BEAS-2B cells

To characterize the inflammatory activation of human lung epithelial cells by *S. pneumoniae*, we infected BEAS-2B cell with pneumococci strain R6x with an infection dose of 10^6 ^cfu/ml. Cytokine release was analyzed using a Bioplex-assay. After 5, 10, and 20 h of incubation, we observed significant induction of MCP-1, GM-CSF, IFNγ, IL-2, IL-4, IL-5, IL-6, IL-8, IL-10, IL-12 (p70), IL-13, IL-17, MIP1β, and TNFα (Fig. 1). IL-1β was found only after 10 and 20 h of infection, and IL-7 level was elevated only after 5 h. Significant time-dependent increase was found for GM-CSF, IFNγ, IL-1β, IL-4, IL-12 (p70), IL-17 and TNFα, whereas IL-6 displayed highest protein level after 5 h of pneumococci exposure.

### *S. pneumoniae *induced c-Jun-NH_2_-terminal kinase-dependent IL-8 release in human lung epithelial BEAS-2B cells

IL-8 is an important chemotactic cytokine in lung inflammation [6] and an established model cytokine for signal transduction analysis [11,25,26] and neutrophil recruitment depended on JNK in different models of acute lung injury [14,27]. In *S. pneumoniae*-infected BEAS-2B cells, we detected JNK2 phosphorylation starting at 30 min post infection (Fig. 2A). After 4 h, pneumococci induced JNK2 phosphorylation similar to TNFα. Inhibition of JNK by specific chemical inhibitor SP600125 dose-dependently reduced *S. pneumoniae*-induced IL-8 protein release (Fig. 2B) and levels of IL-8 mRNA (Fig. 2C) in human lung epithelial BEAS-2B cells. Exemplarily, release of the important inflammatory cytokine IL-6 was also analyzed in cells with inhibited JNK kinase (Fig. 2D). 10 ng/ml of JNK inhibitor SP600125 reduced pneumococci-induced IL-6 release by 50 % (Fig. 2D), while 1 ng/ml had no significant effect (data not shown). Infection with pneumococci or inhibition of JNK with SP600125 did not influence intracellular levels of IL-8 or IL-6 within the timeframe studied (data not shown).

### *S. pneumoniae *induce DNA binding of AP-1 in human lung epithelial BEAS-2B cells

IL-8 gene transcription is in part regulated by JNK-dependent activation of AP-1 in granulocytes [25] as well as lung epithelial cells [28,29]. We found AP-1 DNA-binding after 2, 4, and 7 h of pneumococci infection in human lung epithelial cells (Fig. 3A). 4 h of infection were similar potent in AP-1 activation as 1 h of TNFα stimulation with 10 ng/ml. No activated AP-1 was found 30 min after *S. pneumoniae *infection. Moreover, by using a transcription factor assay kit, we observed DNA binding of phosphorylated AP-1-subunit c-Jun 2 and 4 h after pneumococci-infection of BEAS-2B cells (Fig. 3B). Next we specifically addressed the *il8 *promoter by chromatin immunoprecipitation (ChIP). 2 h after *S. pneumoniae*-infection of human lung epithelial cells, Ser^63/73^-phosphorylated c-Jun and RNA polymerase II (Pol II) were recruited to the endogenous *il8 *promoter (Fig. 3C).

### *S. pneumoniae *induced AP-1-dependent IL-8 release in human epithelial BEAS-2B cells

To verify importance of transcription factor AP-1 on pneumococci-induced IL-8 release, we made use of HEK293 cells transiently transfected with human toll-like receptor 2 (TLR2). After 15 h of pneumococci infection or stimulation with TNFα, IL-8 release could be detected in the supernatant (Fig. 4). HEK293-TLR2 cells were cotransfected with A-Fos (CMV500-A-Fos), a superrepressor of c-Jun-containg AP-1 dimers [19], or empty vector (CMV500). A-Fos strongly reduced pneumococci-, but not TNFα-induced IL-8 release. Antisense constructs for AP-1 subunits Fra1 or Fra2 [20] had no inhibitory effect on IL-8 release by HEK293-TLR2 cells.

## Discussion

Although *S. pneumoniae *is the major pathogen of community-acquired pneumonia [30], little is known about its interaction with target cells, and particularly, with lung epithelial cells [31]. Infection of the human tracheobronchial epithelial cell line BEAS-2B with pneumococci resulted in release of a broad panel of regulatory cyto-, chemokines and growth factors. For example, strong release of the chemoattractants IL-8 (polymorphonuclear neutrophils) and MCP-1 (monocytes) was found. In addition, Th1 cytokines IFNγ and TNFα, as well as Th2 cytokines like IL-4, IL-6, and IL-13 were released after pneumococci infection. Prominent secretion of the pro-inflammatory cytokine IL-1β was observed as well as liberation of myeloid growth factors G-CSF and IL-7. Interestingly, in addition to pro-inflammatory factors, infection of BEAS-2B cells with pneumococci resulted also in production of anti-inflammatory IL-10. This pattern of immunomodulatory factors released by cultured lung epithelial BEAS cells *in vitro *may indicate that activation of tracheobronchial epithelial cells by pneumococci *in vivo *impact on immune reaction in pneumococcal infection. Furthermore, the lung epithelium express membrane bound PRRs (e.g. TLR) [32] as well as cytosolic receptors (e.g. NACHT-LRR protein Nod2) [10], suitable for the detection of invading pneumococci. Taken these facts in consideration, the lung epithelium may function as an important sentinel system for the detection of lung pathogens rather than only comprising a "passive" epithelial barrier.

Thus, we decided to investigate molecular pathways underlying this cytokine response in more detail by using IL-8 as a model cytokine, which is known to be an important chemoattractant in the lung [6]: We observed a time-dependent phosphorylation of JNK – thereby indicating activation – in pneumococci-exposed epithelium. Moreover, JNK inhibition by the chemical inhibitor SP600125 reduced pneumococci-related IL-8 mRNA expression and cytokine release (IL-8, IL-6), while intracellular IL-8 and IL-6 levels remained unchanged. Although other bacteria causing pneumonia, such as *Legionella pneumophila *were shown to activate JNK in human monocytotic cells [33], there are no further studies analyzing JNK activation after infection of pulmonary epithelial cells with bacteria. In rodent models, current studies suggest an important role of JNK for the regulation of lung inflammation besides pneumococci infection. Lipopolysaccharide-related pulmonary neutrophil influx e.g. was limited by inhibition of JNK [34] and this kinase played an important role in ventilation-induced neutrophil infiltration [27]. Moreover, JNK seems to be important for the regulation of the viability of lung epithelium after exposure to active nitrogen species [35]. Although JNK is known to be stimulated by many different types of cellular stress, such as UV, γ -irradiation and pathogen infection, it is reasonable to suggest, that TLR- or Nod-related signaling mediates JNK activation by pneumococci. However, it could not be ruled out that oxidative stress induced by pneumococci-released hydrogen peroxide contributed to JNK activation. Overall, pneumococci-related JNK activation may be an important signaling step in the pneumococci-host interaction process.

Phosphorylation of the transcription factor c-Jun on serine-63 and serine-73 in its N-terminal transactivation domain by activated JNK augments c-Jun transcriptional activity [36,37]. The AP-1 transcription factor is mainly composed of Jun, Fos and ATF protein dimers [38,39]. We found increased AP-1 DNA-binding after pneumococci infection of human lung epithelial cells in EMSA as well as increased DNA binding of phosphorylated AP-1-subunit c-Jun in a specific ELISA demonstrating AP-1 transcription factor activation. Next, we specifically addressed the *il8 *promoter by ChIP and noted recruitment of Ser^63/73^-phosphorylated c-Jun and Pol II to the endogenous *il8 *promoter after *S. pneumoniae*-infection of human lung epithelial cells. In addition, expression of CMV500-A-Fos, a superrepressor of c-Jun-containing AP-1 dimers [19], strongly reduced pneumococci-, but not TNFα-induced IL-8 release verifying the central role of JNK-AP-1 for pneumococci-related IL-8 expression. In contrast to A-Fos, Fra1 and Fra2 proteins – which lack potent transactivation domains – seems not to be involved in pneumococci induced IL-8 expression as evidenced by experiments using antisense constructs for Fra1 or Fra2 [40,41].

However, Tchilibon et al. recently implicated phospho-c-JUN/c-FOS dimers in TNFα-related IL-8 expression in cystic fibrosis lung epithelial cells IB-3 and IB-3/S9 by using MRS2481, a compound inhibiting both signaling of the NF-κB and the AP-1 pathway [42]. In addition, in 16HBE14o-human bronchial epithelial cells TNF-α-induced chemokine expression may be dependent on stimulation of AP-1 pathway [43]. Since our results according the role of the superrepressor CMV500-A-Fos in TNFα-related cell activation were obtained in HEK293 cells, cell- and stimulus-specific effects could not be ruled out.

Overall, pneumococci induced AP-1 activation may contribute significantly to pneumococci-related IL-8 release by pulmonary epithelium.

A central role for JNK in the expression of IL-8 in lung epithelial cells was also reported by Wu et al. who demonstrated JNK-dependent IL-8 expression in the type-II-like alveolar cell line A549 after proteasome inhibition [44]. In addition, stretching of these cells also resulted in JNK-AP-1 dependent IL-8 expression [45]. Although analyzing non-lung cell lines, He et al. provided evidence that severe acute respiratory syndrome (SARS) coronavirus CoV nucleocapsid activated c-Fos suggesting that, besides bacteria, viruses may also induce JNK-AP-1-dependent gene transcription in the lung [46].

However, although cumulating evidence suggests an important role of the JNK-AP-1 signaling pathway in lung inflammation, including pneumococcal pneumonia, several questions remain open. For example, the capability of other important lung pathogens like *Legionella*, viruses or fungi to activate the JNK-AP-1 pathway should be analyzed. *In vivo *experiments addressing the effect of JNK inhibitors in pneumonia models would help to estimate the therapeutic potential of such inhibitors in lung inflammation. Finally, it would be of interest to analyze these signaling pathways in different human primary pulmonary epithelial cells (e.g. small airways, type-I-, type-II-cells).

In this study, we have shown that pneumococci strongly activated secretion of different cytokine families as well as growth factors by BEAS-2B cells. By analyzing IL-8 expression in detail, we demonstrated a central role of the JNK-AP-1 signaling pathway for pneumococci-induced IL-8 liberation by human pulmonary epithelial BEAS-2B cells. Therefore, it is reasonable to suggest that pulmonary epithelial cell could actively participate in immune response in pneumococcal infection.

## Competing interests

The author(s) declare that they have no competing interests.

## Authors' contributions

BS planned the experimental design and drafted the manuscript. KM participated in the study design and performed biochemical and cellular studies. JZ participated in the study design and performed biochemical and cellular studies. VvL participated in the study design and performed molecular studies. PG participated in the study design and performed molecular studies. BO participated in the study design and performed biochemical and cellular studies. SR participated in the study design and performed bacterial studies. NS participated in the study design, helped to draft the manuscript and coordinated the research group. SH participated in the study design, helped to draft the manuscript and coordinated the research group.

The authors declare that they have no competing interests for this study.
